# MOF-Derived ZnSe/N-Doped Carbon Composites for Lithium-Ion Batteries with Enhanced Capacity and Cycling Life

**DOI:** 10.1186/s11671-019-3055-2

**Published:** 2019-07-15

**Authors:** Hongdong Liu, Zongyang Li, Lei Zhang, Haibo Ruan, Rong Hu

**Affiliations:** 1 Engineering Research Center of New Energy Storage Devices and Applications, Chongqing University of Arts and Sciences, Chongqing, 402160 People’s Republic of China; 2 Research Institute for New Materials Technology, Chongqing University of Arts and Sciences, Chongqing, 402160 People’s Republic of China; 3 College of Materials Science and Engineering, Chongqing University, Chongqing, 400045 People’s Republic of China; 4 College of Life Science, Chongqing Normal University, Chongqing, 401331 People’s Republic of China

**Keywords:** Zinc selenide, N-doped carbon, MOFs, Lithium-ion batteries

## Abstract

In this work, three different morphologies of ZnSe/N-doped carbon (NC) composites are synthesized using ZIF-8 by a facile calcination process. By adjusting the particle size of precursor ZIF-8, the morphology and size of the product ZnSe/NC can be controlled. The as-prepared ZnSe/NC composites show excellent cyclic stability and rate capability as anode materials in lithium-ion batteries (LIBs). Especially, the as-obtained ZnSe/NC-300 exhibits reversible discharge capacity of 724.4 mAh g^−1^ after 500 cycles at 1 A g^−1^. The introduction of N-doped carbon can significantly improve the conductivity of ZnSe and promotes the transfer of electrons. And mesoporous structure is conducive to the penetration of electrolyte in active materials, increases the contact area, and alleviates the volume expansion during the charge-discharge process. Thus, ZnSe/NC composites provide a new insight into the development of anode materials for next-generation high-performance LIBs.

**Electronic supplementary material:**

The online version of this article (10.1186/s11671-019-3055-2) contains supplementary material, which is available to authorized users.

## Background

Lithium-ion batteries (LIBs) are widely used as a power source for portable electronic devices and vehicles owing to their high-energy density, long life, and environmental benignity [1–4]. However, present commercial graphite anode materials of LIBs have limited energy capacities and rate performance that are unable to meet the growing needs of high-energy-consuming fields. Lately, transition metal selenides (TMS) have been intensively investigated as anode materials for LIBs to substitute graphite due to their energy density and good cycling performance [5], such as SnSe [6], CoSe [7], Sb_2_Se_3_ [8], MoSe_2_ [9], and FeSe [10]. Among these potential anode materials, zinc selenide (ZnSe) has attracted extensive interest owing to its high theoretical capacity, low cost, and unique electrochemical reaction mechanism [11]. However, ZnSe usually suffers from a large irreversible capacity and poor cycling stability due to a large volume expansion/contraction during Li-ion insertion and extraction process, which results in electrode pulverization and loss of interparticle contact [12, 13]. To overcome these problems, designing nanostructures and combining various carbon materials to alleviate inevitable volume changes and increase conductivity have shown a good prospect for enhancing the electrochemical properties of metal selenides in LIBs. In particular, the N-doped carbon materials greatly change the electronic properties of carbon materials, provide more active sites, improve the interaction between lithium and carbon structure, and enhance the kinetic ability of lithium-ion diffusion and transfer. In addition, the introduction of heteroatoms results in a large number of lattice defects in carbon materials, which can form the disordered carbon structure and further improve lithium storage performance [14–18].

The multifunctional metal-organic frameworks (MOFs) possess many advantages, such as large specific surface area, high porosity, and various structures, and have shown great potential in a wide range of applications, including chemical sensors, gas adsorption/desorption, and catalytic application [19]. Recently, a variety of MOFs have been used as substrates, templates, or sacrificial precursors to fabricate multifunctional nanomaterials for LIBs [20–23]. Especially, TMS composites with carbonaceous materials derived from MOFs not only accelerates Li-ion and electron transport, but also mitigates the large volumetric and structural variation during charge-discharge cycling, thus improving the electrochemical performance of LIBs [24, 25]. For example, Zhu et al. [14] reported ZnSe embedded in N-doped carbon nanocubes derived from ZIF-8 as anode materials for high-performance LIBs. The as-obtained ZnSe/carbon nanocomposite at 600 °C demonstrates a high initial discharge capacity of 1170.8 mAh g^−1^ with the initial columbic efficiency of 68.8% at the current density of 0.1 A g^−1^. After 500 cycles, it still has high reversible capacity.

Herein, we utilized an important member of MOF family, ZIF-8, to synthesize three different morphologies of ZnSe/N-doped carbon (NC) composites by a facile calcination process. The resulting composites present excellent cyclic stability and rate capability as anode materials in LIBs. Particularly, the as-prepared ZnSe/NC-300 exhibits reversible discharge capacity of 724.4 mAh g^−1^ after 500 cycles at 1 A g^−1^. Hence, ZnSe/NC nanocomposites show outstanding electrochemical performance, which will be a potential high-performance anode material for LIBs.

## Methods

### Material Preparation

#### Synthesis of ZIF-8 Precursors

In a typical process, ZIF-8 was prepared by the commonly liquid phase method. Zn(NO_3_)_2_·6H_2_O and 2-methylimidazole were used as raw materials and methanol was used as solvent. Firstly, 25 mmol of 2-methylimidazole and a certain amount of (0, 0.22, 0.44 mmol) Zn(NO_3_)_2_·6H_2_O were dissolved in 250 ml methanol to form A solution and 12.5 mmol of Zn(NO_3_)_2_·6H_2_O was dissolved in 250 ml methanol to obtain B solution. After the solution was completely dissolved, the solution B was poured into the solution A and exposed to ultrasound for 10 min. Subsequently, the mixed solutions were kept at room temperature for 24 h. After the reaction, the products were centrifugally washed many times with methanol and then dried in a vacuum drying chamber at 60 °C for 12 h. ZIF-8 with diameters of 900, 300, and 40 nm can be obtained, named ZIF-8-900, ZIF-8-300, and ZIF-8-40, respectively.

#### Synthesis of ZnSe/NC Composites

The as-prepared ZIF-8 was mixed with selenium powders according to the mass ratio of 1:1. The powders were mixed with a mortar and placed in a high-temperature tubular furnace. The ZnSe/NC composites were obtained 800 °C for 4 h under argon atmosphere. Both of the heating rate and cooling rate were 2 °C/min. The composites were named as ZnSe/NC-40, ZnSe/NC-300, and ZnSe/NC-900, respectively. In addition, the commercial ZnSe was used as control group for comparative experiments.

### Material Characterization

X-ray power diffraction (XRD) patterns were obtained from TD-3500 X-ray diffractometer equipped with Cu/Ka radiation (*λ* = 0.15406 nm) between 10° and 80° with a scanning rate of 4°min^−1^. Raman spectra were employed with a micro-Raman spectrometer (LabRAM HR800) at a wave length of 633 nm (1.96 eV). Specific surface area and pore size distribution were determined by a Belsorp II analyzer through the Brunauer-Emmett-Teller (BET) method and Barrett-Joyner-Halenda (BJH) model. The structure and morphology of ZnSe/NC were observed by field emission scanning electron microscope (FEI Quanta 250) and transmission electron microscopy (TEM-FEI Tecnai G2 F20). The main element composition of ZnSe/NC-300 was carried on X-ray photoelectron spectroscopy (XPS; Thermo VG ESCALAB 250XI).

### Electrochemical Measurements

The electrochemical properties of ZnSe/NC composites and commercial ZnSe were investigated using coin-type cells (CR2032). Working electrodes were comprised of 80 wt.% active materials (ZnSe/NC-900, ZnSe/NC-300, ZnSe/NC-40, or commercial ZnSe), 10 wt.% acetylene black, and 10% wt.% polyvinylidene fluoride (PVDF). These materials were dispersed in *N*-methy-2-pyrrolidone (NMP) to produce homogeneous slurry. Subsequently, the resultant slurry was coated uniformly onto a 10-μm-thick Cu foil using scraper technology and then dried in a vacuum oven for 8 h at 120 °C. A pure lithium sheet was used as counter electrode. The electrolyte was 1 M LiPF_6_ (1.0 M) with the mixture of ethylene carbonate (EC) and dimethyl carbonate (DMC) (1:1 *v*/*v*). The polypropylene membrane (Celgard2400) served as the separator to electronically separate the two electrodes. The CR2032-type coin cells were assembled in an Ar-filled glove box. Galvanostatic cycling measurements were performed on a Neware battery test system (BTS-610) at various densities between 0.01 and 3.0 V. Cyclic voltammetry (CV) and electrochemical impedance spectroscopy (EIS) were carried out using a CHI760E electrochemical workstation. The scanning rate of CV was 0.2 mV/s at the range of 0.01–3.0 V, and the frequency range of EIS was between 0.1 Hz and 100 kHz.

## Results and Discussion

**Fig. 1:**
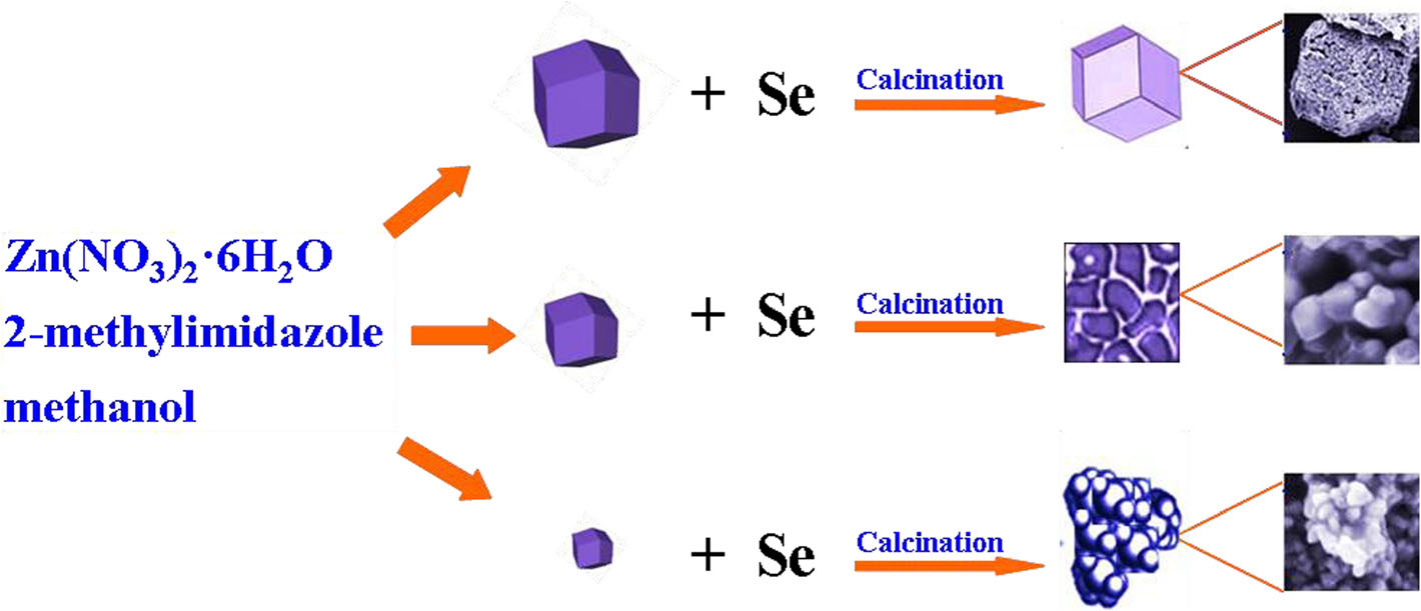
Schematic illustration of the fabrication process of ZnSe/NC composites

Figure 1 illustrates the fabrication process of three different morphologies of ZnSe/NC composites by a facile chemical precipitation-calcination method. Firstly, the precursor ZIF-8 with different particle sizes are synthesized by dissolving different quantities of Zn(NO_3_)_2_·6H_2_O and 2-methylimidazole in methanol for a period of time to form precipitation. Among them, Zn(NO_3_)_2_·6H_2_O provides zinc source, and 2-methylimidazole provides carbon source and nitrogen source. The crystallization of MOF(ZIF-8) consists of two processes, nucleation and nucleation growth, which often proceed simultaneously and determine the crystal size together. Rapid nucleation is beneficial to the reduction of crystal size. Therefore, the crystal size of ZIF-8 can be significantly reduced by adding a small amount of metal ions in advance and then adding a large number of metal ions to make the nucleation grow up. Then, the as-prepared ZIF-8 and selenium powders are mixed in a certain proportion and calcined at high temperature in argon atmosphere to obtain ZnSe/NC composites. By adjusting the particle size of precursor ZIF-8, the morphology and size of the product ZnSe/NC can be controlled.

**Fig. 2:**
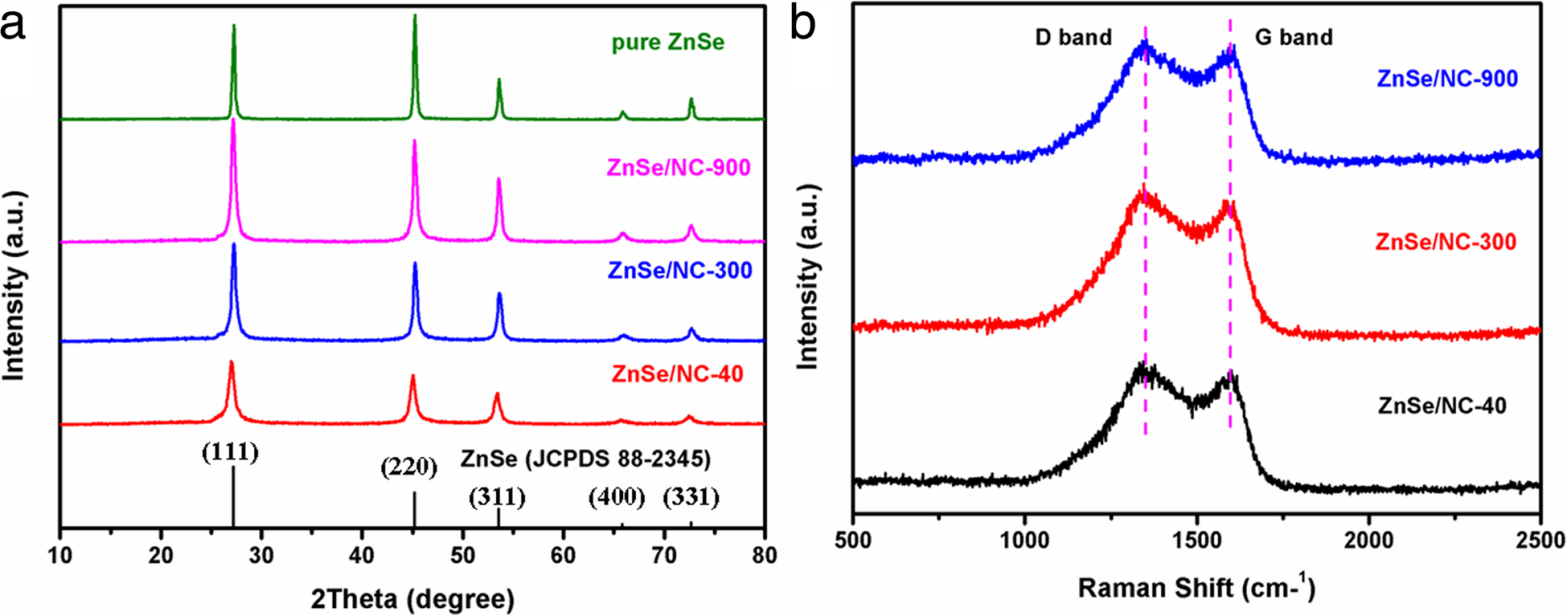
**a** The XRD patterns of pure ZnSe, ZnSe/NC-900, ZnSe/NC-300, and ZnSe/NC-40. **b** Raman spectrum of ZnSe/NC-900, ZnSe/NC-300, and ZnSe/NC-40

The XRD patterns of the samples are shown in Fig. 2a. The XRD spectra of pure ZnSe, ZnSe/NC-900, ZnSe/NC-300, and ZnSe/NC-40 are consistent with the standard spectra of ZnSe (JCPDS 88-2345). The sharp diffraction peaks indicate that the as-prepared ZnSe/NC composites have high crystallinity. Moreover, the intensity of diffraction peaks of ZnSe/NC-40, ZnSe/NC-300, and ZnSe/NC-900 increase gradually, which indicates that the grain size of ZnSe phase increases, because ZnSe/NC inherits the grain size of precursor ZIF-8 to a certain extent. And no graphite-carbon bulges are found near 2*θ* = 24°, which may be related to the lower content of C in ZnSe/NC composites and the existence form of C.

Raman spectra of ZnSe/NC-900, ZnSe/NC-300, and ZnSe/NC-40 were measured to investigate the existence of carbon and the form of carbon in ZnSe/C composites, respectively, as shown in Fig. 2b. Three samples of ZnSe/NC have a wide peak at about 1350 cm^−1^ and 1597 cm^−1^, corresponding to the D-band and G-band vibrations of carbon, respectively. D peak is usually considered to be caused by disordered vibration of defects in carbon materials, while G peak is caused by in-plane stretching vibration of carbon atoms with sp2, which is the characteristic peak of graphite carbon [26]. The existence of D and G peaks indicates that carbon exists in ZnSe/NC, which is formed by carbonization of organic ligand 2-methylimidazole in ZIF-8 at high temperature. The ID/IG values of ZnSe/NC-900, ZnSe/NC-300, and ZnSe/NC-40 are 1.03, 1.04, and 1.02, respectively. The ID/IG values of the three composites are relatively close and large, which indicates that the graphitization degree of carbon in ZnSe/NC composites is low and there are many defects. According to the relevant literature [27], defects in carbon materials can be used as residual lithium storage reaction of active sites to increase capacity.

**Fig. 3:**
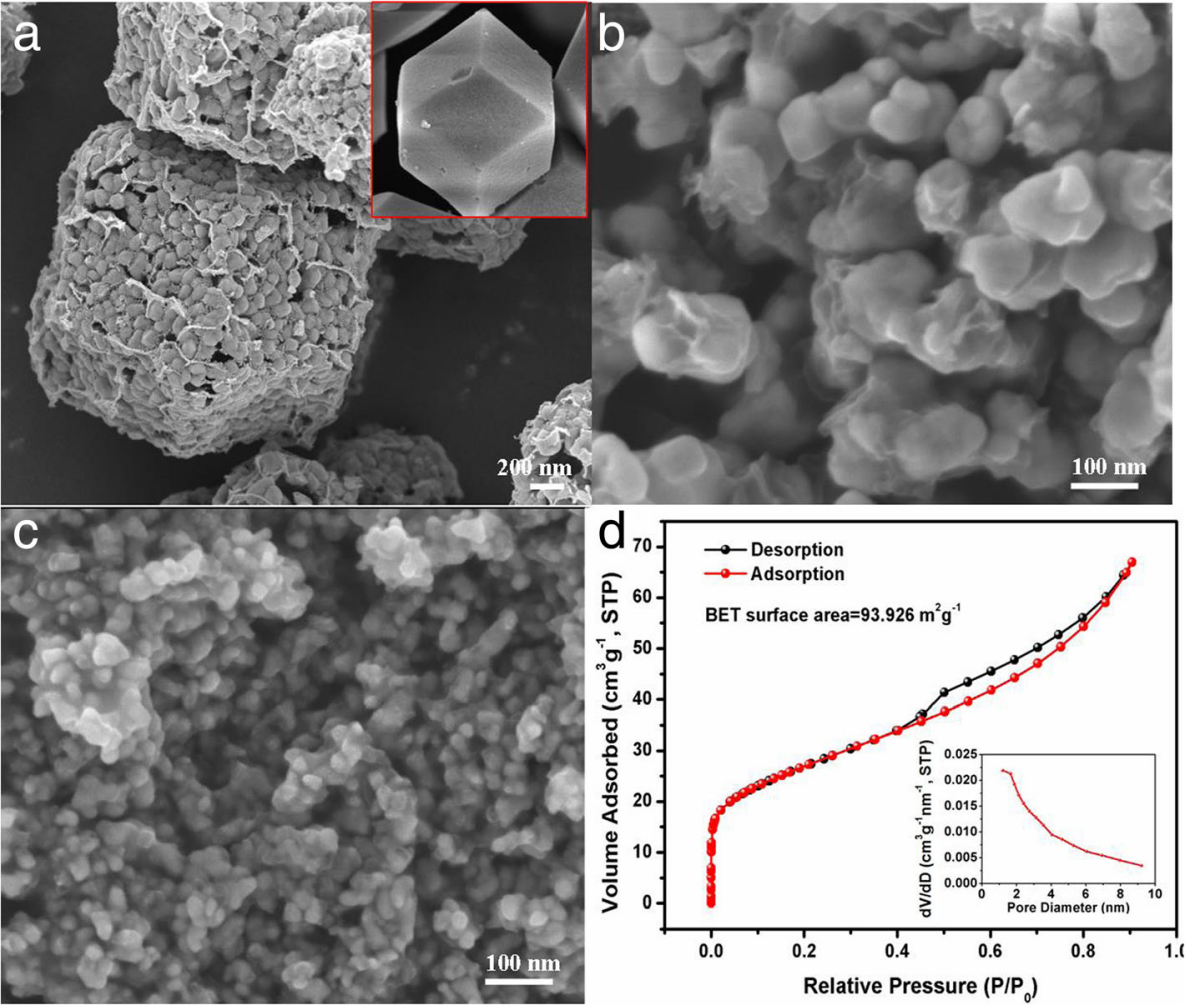
**a**–**c** SEM images of ZnSe/NC-900, ZnSe/NC-300, and ZnSe/NC-40, respectively, inset of SEM image of ZIF-8. **d** Nitrogen adsorption-desorption isotherms of ZnSe/NC-300, inset of pore diameter distribution profiles of ZnSe/NC-300

The morphologies of ZnSe/NC-900, ZnSe/NC-300, and ZnSe/NC-40 were measured by FESEM, as shown in Fig. 3a–c. The rhombic dodecahedron morphology of ZnSe/NC-900 can be observed in Fig. 3a; the inset shows the SEM image of precursor ZIF-8. And the dodecahedron is composed of numerous ZnSe nanoparticles of about 100 nm in size, and the outer layer of the whole dodecahedron is coated with a thin carbon layer. However, ZnSe/NC-300 cannot maintain the dodecahedron morphology, but shows nanoparticles with a particle size of about 20–50 nm, and ZnSe is encapsulated by carbon layers. ZnSe/NC-40 is also a nanoparticle with a particle size of about 10–20 nm and carbon layers wrapped in the outer layer, but its agglomeration is serious. Figure 3d shows the BET curve of ZnSe/NC-300. It can be observed that the nitrogen adsorption and desorption curve of ZnSe/NC-300 composites have obvious hysteresis loops in the range of relative pressure 0.5–0.9 p/p0, indicating that they are type IV isothermal curves. At the same time, the hysteresis loop is H3 type, which indicates the existence of mesoporous structure in ZnSe/NC composites. According to Brunauer-Emmett-Teller (BET) theory, the specific surface area of ZnSe/C-300 is 93.926 m^2^ g^−1^. The inset of Fig. 3d is a pore diameter distribution curve based on Barrett-Joyner-Halenda (BJH) theory. The average pore size of ZnSe/NC-300 is 4.4095 nm, which is typical mesoporous structure. According to the relevant literature [28], mesoporous structure is conducive to the penetration of electrolyte in the active materials, increases the contact area between the electrolyte and the active materials, enlarges the reactive sites, and promotes the diffusion of lithium ions. Furthermore, mesoporous structure can also alleviate the volume expansion and stress during charge-discharge process and improves cycle stability.

**Fig. 4:**
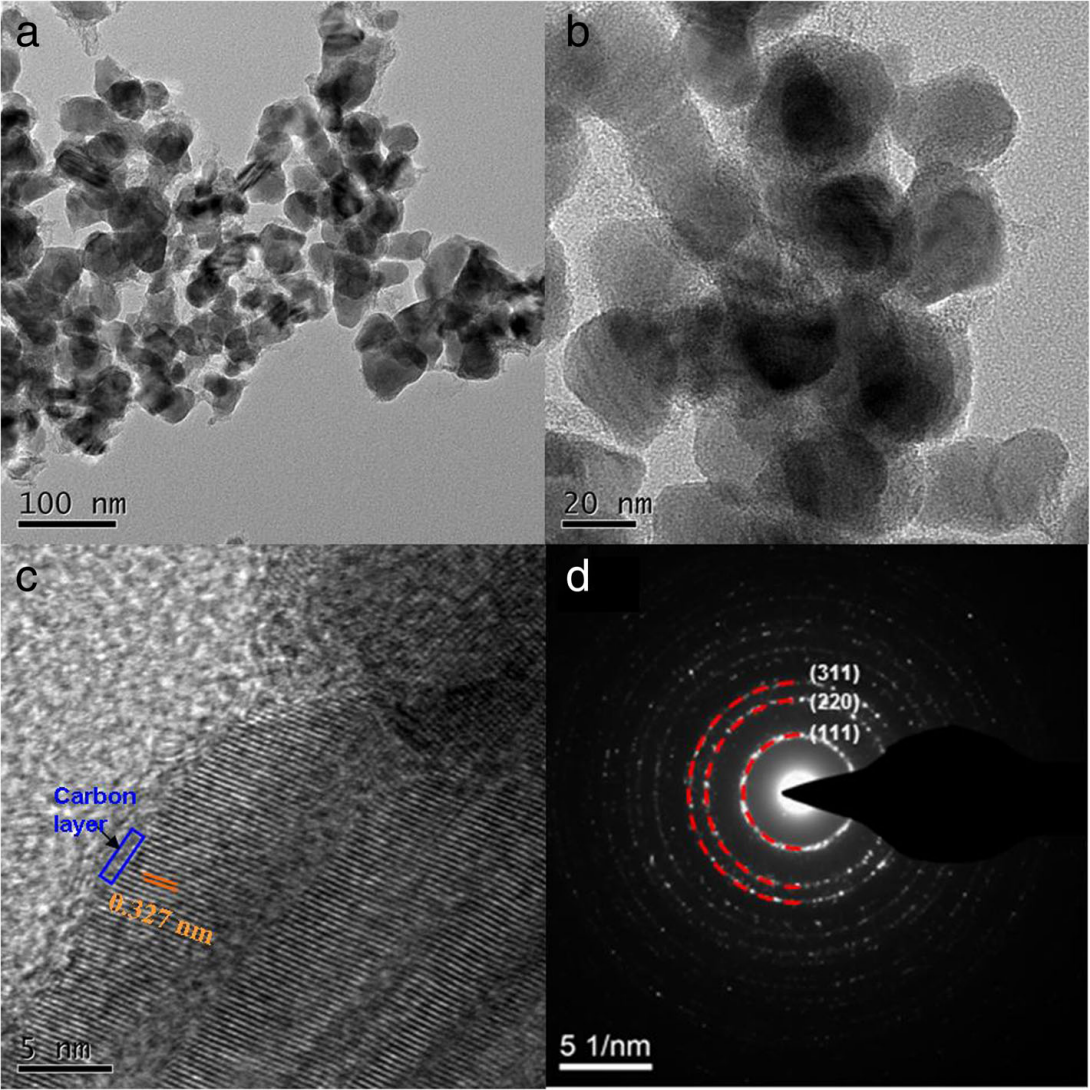
**a**, **b** TEM images of ZnSe/NC-300. **c** HRTEM image of ZnSe/NC-300. **d** SAED image of ZnSe/NC-300

The morphology and crystal structure of ZnSe/NC-300 were further characterized by TEM. ZnSe/NC-300 cannot inherit the rhombohedral dodecahedron morphology of the precursor ZIF-8, but shows nano-granular architecture with particle size of about 20–50 nm in Fig. 4a, b. Figure 4c is a HRTEM image of ZnSe/NC composites, from which uniform carbon layers and lattice fringes can be clearly seen. The crystal plane spacing in ZnSe/NC-300 is 0.33 nm, corresponding to (111) crystal plane of ZnSe. This result is consistent with the XRD and XPS. Figure 4d is the selected area electron diffraction pattern of ZnSe/NC-300. It can be seen that the patterns of electron diffraction are all diffraction rings, not uniform diffraction spots. It shows that the as-prepared ZnSe/NC-300 composites are polycrystalline.

**Fig. 5:**
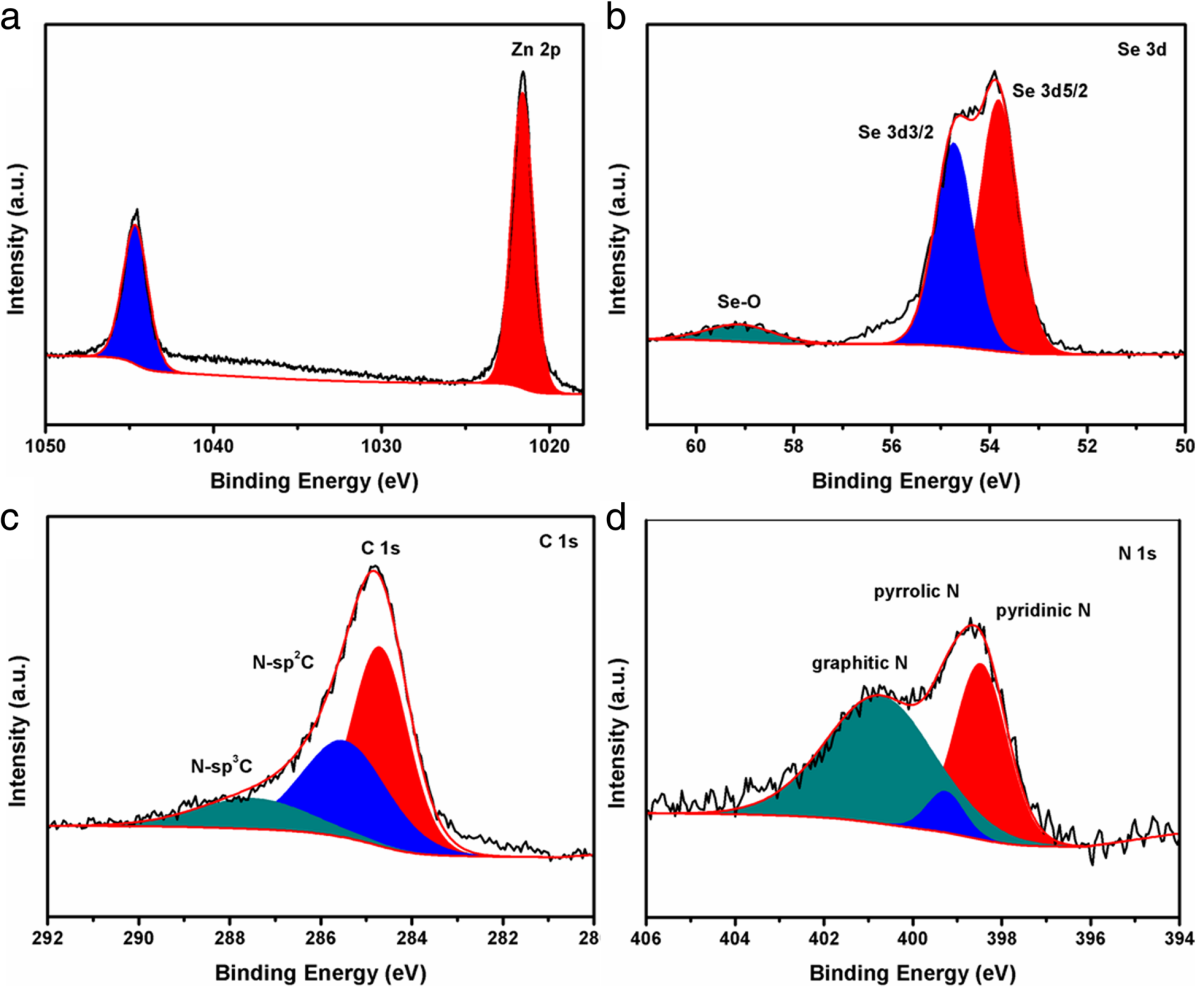
High-resolution XPS spectra of ZnSe/NC-300 **a** Zn 2p, **b** Se3d, **c** N 1s, and **d** C 1s

XPS spectra of ZnSe/C-300 composites were measured for further analysis of the element composition and the existing state of each element. The characteristic peaks of Zn (Zn 2p), Se (Se 3 s, Se 3P and Se 3d), C (C 1 s), N (N1 s), and O (O 1 s) can be seen from the full spectrum of XPS (Additional file 1: Figure S3), which indicates that Zn, Se, C, N, and O are five elements in ZnSe/C-300. N may be derived from organic ligand 2-methylimidazole, which is carbonized to form N-doped carbon during high-temperature calcination. The O 1s peak can be associated with the adsorbed O2, CO2, and H2O in the air or the surface oxidation of the samples [14]. Figure 5 a–d are Zn 2p, Se 3d, C 1s, and N1s high-resolution XPS spectra of ZnSe/NC-300, respectively. From Fig. 5a, it can be seen that there are two characteristic peaks at 1044.65 eV and 1021.62 eV in the spectrum of Zn 2p, corresponding to Zn 2p1/2 and Zn 2p3/2 in ZnSe, respectively, and the difference of binding energy value between the two peaks is Δ*E* = 23.03 eV, which indicates that Zn in the ZnSe/NC-300 mainly exists in the form of Zn^+2^. Three characteristic peaks can be seen from the high-resolution spectra of Fig. 5b Se 3d. The peaks at 54.7 eV and 53.82 eV correspond to Se 3d3/2 and Se 3d5/2, while the broad peak at 59.09 eV corresponds to Se-O; it may be that the exposure of ZnSe/NC-300 to air results in the oxidation of the surface layer to SeO_*x*_. Figure 5c is a high-resolution C 1s spectrum of ZnSe/NC-300, from which three characteristic peaks at 284.7 eV, 285.49 eV, and 287.48 eV can be seen, corresponding to sp2C, N-sp2C, and N-sp3C, respectively. In Fig. 5d N 1s, there are three characteristic peaks located at 400.74 eV, 399.26 eV, and 398.47 eV, corresponding to graphite nitrogen, pyrrolidine nitrogen, and pyridine nitrogen, respectively. According to the relevant literature [29], pyrrolidine and pyridine nitrogen can be used as active sites to participate in lithium storage reaction and improve the capacity of materials. In addition, N doping can provide enough electrons for the *π* conjugate system and further improves its conductivity [30–33]. Therefore, it can be seen that ZnSe/NC-300 is composed of ZnSe and N-doped carbon, which is consistent with the results of XRD and Raman.

**Fig. 6:**
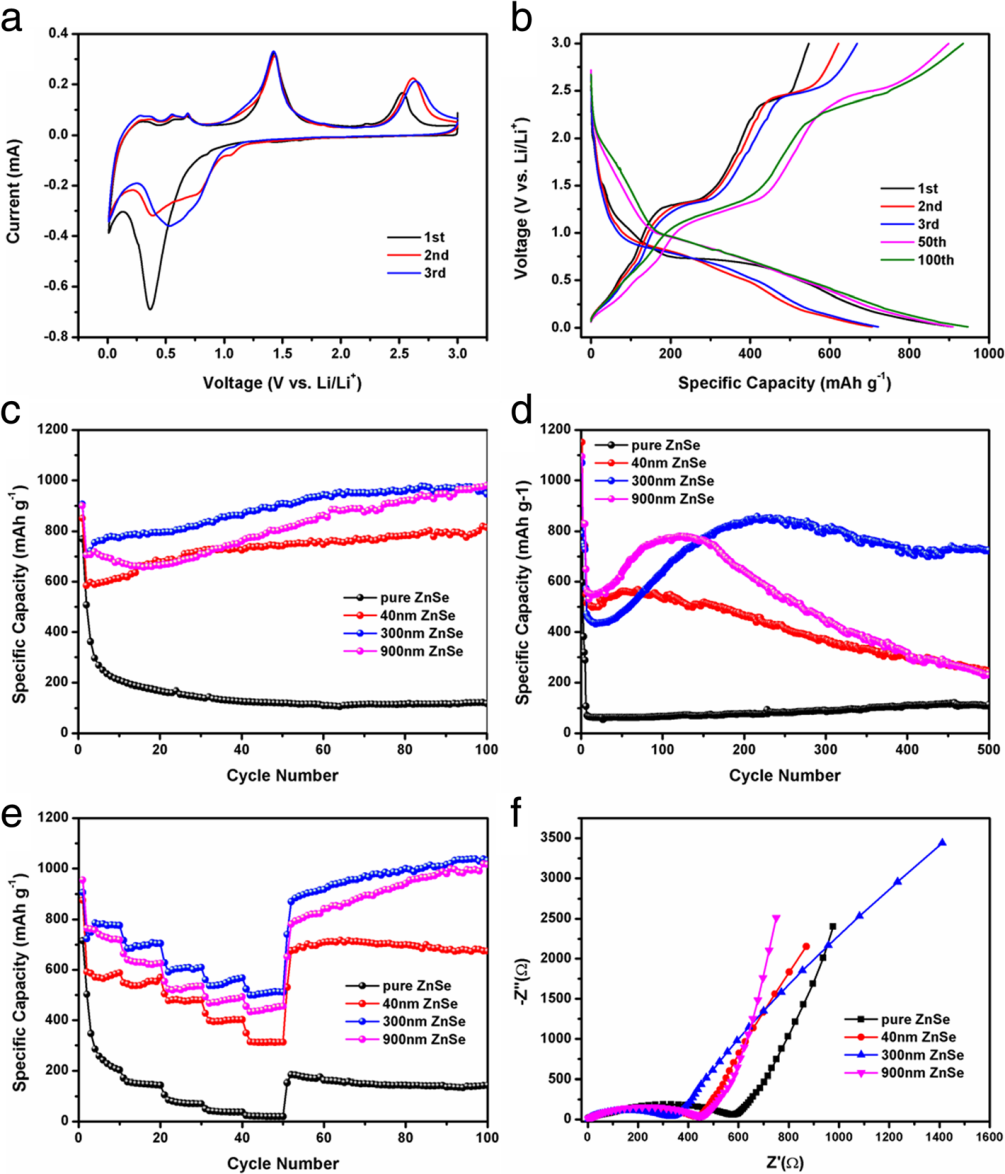
**a** The first three cyclic CV cures of ZnSe/NC-300. **b** Galvanostatic charge-discharge curves at the current density of 100 mA g^−1^. **c** The cycling performance of pure ZnSe, ZnSe/NC-900, ZnSe/NC-300, and ZnSe/NC-40 at the current density of 100 mA g^−1^. **d** The cycling performance of pure ZnSe, ZnSe/NC-900, ZnSe/NC-300, and ZnSe/NC-40 at the current density of 1 A g^−1^. **e** Rate performance of pure ZnSe, ZnSe/NC-900, ZnSe/NC-300, and ZnSe/NC-40 at the current densities ranging from 0.1 to 2 A g^−1^. **f** EIS spectra of pure ZnSe, ZnSe/NC-900, ZnSe/NC-300, and ZnSe/NC-40 before cycling

In order to investigate the lithium storage mechanism of ZnSe/NC composites, the CV curves of ZnSe/NC-300 composites were measured. As shown in Fig. 6a, there is a weak reduction peak at 1.5 V and a sharp reduction peak at 0.35 V in the first discharge process of ZnSe/NC composites. According to previous reports [11, 29], the reduction peak at 1.5 V is caused by the formation of SEI film on the surface of active materials. The reduction peak at 0.35 V indicates that lithium ion is embedded in the crystal structure of ZnSe, ZnSe is reduced to form Zn and Li_2_Se, and Zn and Li-ion are alloyed to form Li_*x*_Zn alloy phase. In the first charging process, there are several small oxidation peaks below 1.0 V, which correspond to the multi-step de-alloying reaction of Li_*x*_Zn alloy phase. The sharp oxidation peak at about 1.4 V corresponds to the formation of ZnSe by oxidation of Zn. In addition, several oxidation peaks can be observed at about 2.5 V for ZnSe/NC composites, which may be related to the oxidation of Li_2_Se to Se. In the following two cycles, the reduction and oxidation peaks of ZnSe/NC composites deviate from those of the first cycle, which may be caused by the restructuring of ZnSe/NC composites during charging and discharging.

Figure 6b shows the galvanostatic charge-discharge curve of ZnSe/NC-300 measured at the current density of 100 mA g^−1^ and voltage window of 0.01–3.0 V. It can be seen that there is a discharge platform at about 0.75 V in the first discharge process of ZnSe/NC composites. During the first charging process, there is a charging platform around 1.3 V. They correspond to the insertion and removal of lithium ions, respectively. During the subsequent charge-discharge process, the discharge platform changes from 0.75 to 0.9 V, while the charge platform does not change significantly. The results are in good agreement with those of CV curves.

The first charge-discharge capacities of ZnSe/NC-300 are 547.48 mAh g^−1^ and 906.66 mAh g^−1^, respectively. The initial coulomb efficiency is 60.3%. The lower coulomb efficiency and irreversible capacity in the first cycle are caused by the irreversible decomposition of electrolytes on the surface of active materials to form SEI films. Moreover, the charge-discharge curves of the 50th and 100th cycles are also shown in Fig. 6b. It can be found that the discharge capacities of the 50th and 100th cycles of ZnSe/NC composites increase significantly compared with the first three cycles, which may be caused by the pseudo-capacitance behavior.

Cycle performances of ZnSe/NC composites were further investigated at the current densities of 100 mA g^−1^. It can be seen from Fig. 6c that the discharge capacity of ZnSe/NC composites shows an increasing trend. After 100 cycles, the discharge capacity of ZnSe/NC-900 increases from 705.85 mAh g^−1^ in the second cycle to 979.15 mAh g^−1^. The corresponding capacity of ZnSe/NC-300 increases from 706.05 to 947.11 mAh g^−1^. The capacity of ZnSe/NC-40 rises from 584.58 to 814.6 mAh g^−1^. According to the previous literature [14], this capacity growth phenomenon is caused by the pseudo-capacitance behavior. The pseudo-capacitance is due to the formation of a highly reversible gel polymer layer on the surface of the active materials. This reaction includes the oxidation-reduction reaction on the surface and near the surface of active materials and the rapid insertion of particles. The capacity growth phenomenon caused by pseudo-capacitance is common in transition metal compounds which store lithium through conversion reaction.

In addition, the discharge capacity of ZnSe/NC composites is much higher than that of pure ZnSe in the whole cyclic testing process. This is because that the introduction of N-doped carbon can significantly improve the conductivity of ZnSe and promotes the transfer of electrons. At the same time, pyrroe-N and pyridine-N can be used as reactive sites to participate in lithium storage reaction and improve lithium storage capacity. Furthermore, mesoporous structure is conducive to the penetration of electrolyte in active materials, increases the contact area, and alleviates the volume expansion during the charge-discharge process.

Figure 6d shows the cyclic behavior of ZnSe/NC composites at a high current density of 1 A g^−1^. The first five cycles are carried out at a current density of 100 mA g^−1^. The purpose is to generate a dense SEI film on the surface of the active materials for the subsequent cycling performance test at the high current density of 1 A g^−1^. The discharge capacity of ZnSe/NC composites shows a trend of first increasing and then decreasing. The discharge capacity of ZnSe/NC-300 increases to 858.05 mAh g^−1^ in the 216th cycle and decreases to 724.4 mAh g^−1^ in the 500th cycle. It is significantly superior than that of the previous literatures (Additional file 1: Table S1). The discharge capacity of ZnSe/NC-900 rises to 779.86 mAh g^−1^ in the 121st cycle and reduces to 229.54 mAh g^−1^ in the 500th cycle. The capacity of ZnSe/NC-40 improves to the maximum at the 70th cycle and drops to 243.27 mAh g^−1^ at the 500th cycle.

Figure 6e shows the rate performance curves of pure ZnSe, ZnSe/NC-900, ZnSe/NC-300, and ZnSe/NC-40 (0.1–2 A). After 10 cycles at the current densities of 100 mAh g^−1^, 200 mA g^−1^, 500 mA g^−1^, 1 A g^−1^, and 2 A g^-1^, respectively, the corresponding discharge capacities of ZnSe/NC-300 are 775.65 mAh g^−1^, 704.14 mAh g^−1^, 609.26 mAh g^−1^, 567.68 mAh g^−1^, and 511.59 mAh g^−1^. The corresponding discharge capacities of ZnSe/NC-900 are 718.59 mAh g^−1^, 625.73 mAh g^−1^, 534.94 mAh g^−1^, 492.61 mAh g^−1^, and 455.28 mAh g^−1^, respectively. The discharge capacities of ZnSe/C-40 are 587.73 mAh g^−1^, 569.35 mAh g^−1^, 479.64 mAh g^−1^, 402.31 mAh g^−1^, and 312.57 mAh g^−1^, respectively. In addition, as the current density decreases to 100 mA g^−1^, the discharge capacity of ZnSe/NC-40 remains basically stable, while the discharge capacity of ZnSe/NC-300 and ZnSe/NC-900 shows an increasing trend. When the current density drops to 100 mAh g^−1^, the discharge capacity of ZnSe/NC-300 recovers to 739.89 mAh g^−1^, and increases to 1031.66 mAh g^−1^ after 50 cycles. The discharge capacity of ZnSe/NC-900 is restored to 651.97 mAh g^−1^, and raised to 1016.07 mAh g^−1^ after 50 cycles. The above results show that the structure of ZnSe/NC composites has not been damaged obviously after the rate performance test, and the integrity of the structure has been maintained, presenting a good rate performance. Compared with ZnSe/NC-300 and ZnSe/NC-900, the rate performance of ZnSe/NC-40 is worse. This may be due to its smaller specific surface area (Additional file 1: Figure S4), which reduces the contact area between ZnSe/NC and electrolyte, and is not conducive to the diffusion of lithium ions.

The impedance of the material has a significant effect on its electrochemical properties. Figure 6f shows the AC impedance spectra of pure ZnSe and ZnSe/NC composites before cyclic testing. It can be seen that the AC impedance spectra consist of a half circle in the high-frequency region and an oblique line in the low-frequency region. The diameter of the semicircle represents the charge transfer resistance, while the slope of the oblique line presents the Warburg impedance, which is related to the diffusion of lithium ions in the electrode materials. The semicircle diameter of ZnSe/NC composite is obviously smaller than that of pure ZnSe, which indicates that the charge transfer impedance of ZnSe/NC decreases because the introduction of N-doped carbon promotes the electron transfer and reduces the resistance. The AC impedance spectroscopy (Additional file 1: Figure S7) of ZnSe/C composites after 100 cycles shows that the semicircle diameter of the composites at high frequency decreases significantly, which may be related to the activation behavior during the cyclic process.

## Conclusions

In summary, three different morphologies of ZnSe/NC composites have been synthesized by using ZIF-8 nanocubic templates followed by a facile calcination method. By adjusting the particle size of precursor ZIF-8, the morphology of the product ZnSe/NC can be controlled. As a result, ZnSe/NC composites exhibit excellent cyclic stability and rate capability as anode materials in LIBs. Particularly, the as-obtained ZnSe/NC-300 presents that the first discharge and charge capacity are 906.66 and 547.48 mAh g^−1^ at the current density of 100 mA g^−1^, respectively. After 500 cycles, the reversible discharge capacity is still maintained at 724.4 mAh g^−1^ at 1 A g^−1^. The introduction of N-doped carbon can significantly improve the conductivity of ZnSe and promotes the transfer of electrons. And the large specific surface area and mesoporous structure are conducive to the penetration of electrolyte in active materials, increase the contact area, and alleviate the volume expansion during the charge-discharge process. Therefore, the as-prepared ZnSe/NC nanocomposites show superior electrochemical performance, which will be a potential high-performance anode material for LIBs.

## Additional file

Additional file 1: **Figure S1.** SEM images of ZIF-8 at different sizes (a) ZIF-900, (b) ZIF-300, (c) ZIF-40, and (d) the XRD patterns of synthesized ZIF-8 at different sizes and simulated XRD pattern. **Figure S2.** (a, b) TEM images of ZnSe/NC-900 and ZnSe/NC-40, respectively, (c, d) HRTEM images of ZnSe/NC-900 and ZnSe/NC-40, respectively, (e, f) SAED images of ZnSe/NC-900 and ZnSe/NC-40, respectively. **Figure S3.** XPS survey spectra of ZnSe/NC-300. **Figure S4.** (a, b) Nitrogen adsorption-desorption isotherms of ZnSe/NC-900 and ZnSe/NC-40, respectively, (c, d) their pore diameter distribution profiles. **Figure S5.** The first three cyclic CV cures of (a) pure ZnSe, (b) ZnSe/NC-900, and (c) ZnSe/NC-40 at a scan rate of 0.2 mV/s in the range of 0.01–3.0 V. **Figure S6.** Galvanostatic discharge/charge voltage profiles of (a) pure ZnSe, (b) ZnSe/NC-900, (c) ZnSe/NC-40 at a current density of 100 mA g^−1^. **Figure S7.** EIS spectra of pure ZnSe, ZnSe/NC-900, ZnSe/NC-300, and ZnSe/NC-40 after 100 cycles. **Table S1.** Comparison of ZnSe/NC composites and other metal selenides as LIB anodes. (DOC 2425 kb)

## Data Availability

All data are fully available without restriction.

## Abbreviations

LIBs: Lithium-ion batteries
NC: N-doped carbon
TMS: Transition metal selenides
MOFs: Metal-organic frameworks
XRD: X-ray power diffraction
BET: Brunauer-Emmett-Teller
BJH: Barrett-Joyner-Halenda
TEM: Transmission electron microscopy
PVDF: Polyvinylidene fluoride
EC: Ethylene carbonate
DMC: Dimethyl carbonate
CV: Cyclic voltammetry
EIS: Electrochemical impedance spectroscopy
