# Supplementary material for: MOF-Derived ZnSe/N-Doped Carbon Composites for Lithium-Ion Batteries with Enhanced Capacity and Cycling Life
Source: Nanoscale Res Lett. 2019 Jul 15;14:237. doi: 10.1186/s11671-019-3055-2 (PMC6629758; doi:10.1186/s11671-019-3055-2)
Supplement: Supplementary file 1 — Figure S1. SEM images of ZIF-8 at different sizes (a) ZIF-900, (b) ZIF-300, (c) ZIF-40, and (d) the XRD patterns of synthesized ZIF-8 at different sizes and simulated XRD pattern. Figure S2. (a, b) TEM images of ZnSe/NC-900 and ZnSe/NC-40, respectively, (c, d) HRTEM images of ZnSe/NC-900 and ZnSe/NC-40, respectively, (e, f) SAED images of ZnSe/NC-900 and ZnSe/NC-40, respectively. Figure S3. XPS survey spectra of ZnSe/NC-300. Figure S4. (a, b) Nitrogen adsorption-desorption isotherms of ZnSe/NC-900 and ZnSe/NC-40, respectively, (c, d) their pore diameter distribution profiles. Figure S5. The first three cyclic CV cures of (a) pure ZnSe, (b) ZnSe/NC-900, and (c) ZnSe/NC-40 at a scan rate of 0.2 mV/s in the range of 0.01–3.0 V. Figure S6. Galvanostatic discharge/charge voltage profiles of (a) pure ZnSe, (b) ZnSe/NC-900, (c) ZnSe/NC-40 at a current density of 100 mA g−1. Figure S7. EIS spectra of pure ZnSe, ZnSe/NC-900, ZnSe/NC-300, and ZnSe/NC-40 after 100 cycles. Table S1. Comparison of ZnSe/NC composites and other metal selenides as LIB anodes. (DOC 2425 kb) [file 11671_2019_3055_MOESM1_ESM.doc]

**MOFs derived ZnSe/N-doped carbon composites for lithium-ion batteries with enhanced capacity and cycling life**

Hongdong Liu^1,2^, Zongyang Li^3^, Lei Zhang^4*^, Haibo Ruan^2^, Rong Hu^2*^

^1^ Engineering research center of new energy storage devices and applications, Chongqing University of Arts and Sciences, Chongqing 402160, PR China

^2^ Research institute for new materials technology, Chongqing University of Arts and Sciences, Chongqing 402160, PR China

^3^ College of Materials Science and Engineering, Chongqing University, Chongqing 400045, PR China

^4^ College of life science, Chongqing Normal University, Chongqing 401331, PR China

lhd0415@126.com(H.L.); 710433359@qq.com(Z.L.); rhbcqu@aliyun.com (H.R.)

***** Correspondence: leizhang0215@126.com(L.Z.); hurong_82@163.com (R.H.)


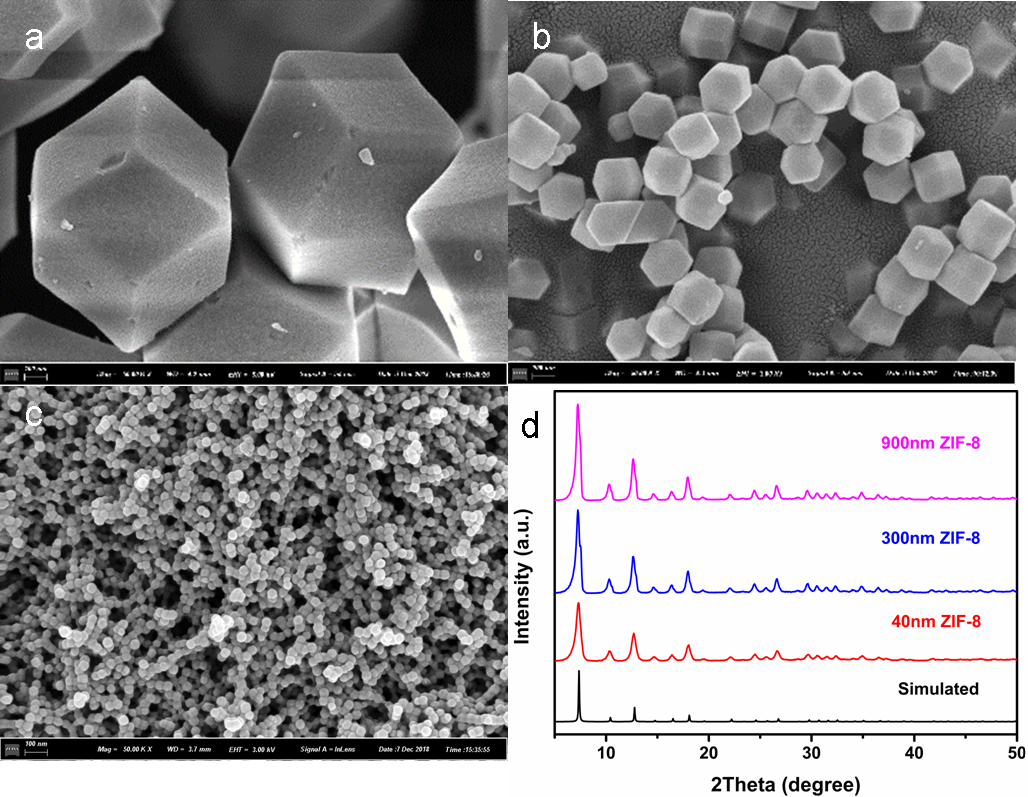


Additional file 1: Figure S1. SEM images of ZIF-8 at different sizes (a) ZIF-900, （b）ZIF-300, (c) ZIF-40 and (d) the XRD patterns of synthesized ZIF-8 at different sizes and simulated XRD pattern.


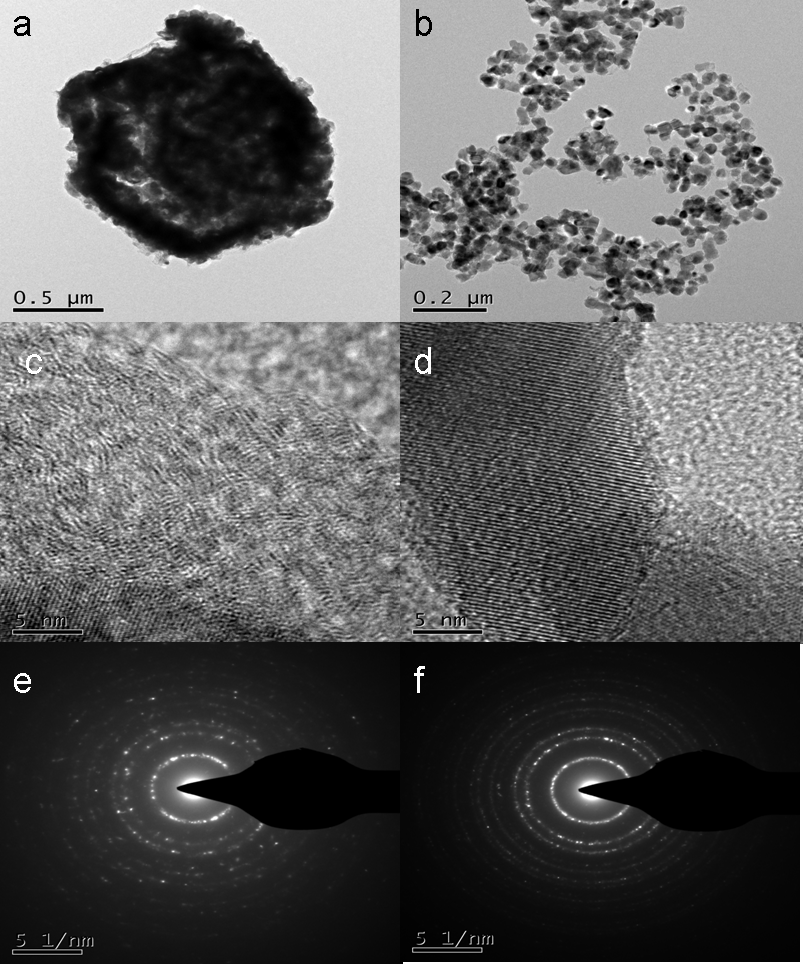


Additional file 1: Figure S2.（a,b）TEM images of ZnSe/NC-900 and ZnSe/NC-40, respectively,（c,d）HRTEM images of ZnSe/NC-900 and ZnSe/NC-40, respectively,（e,f）SAED images of ZnSe/NC-900 and ZnSe/NC-40, respectively.


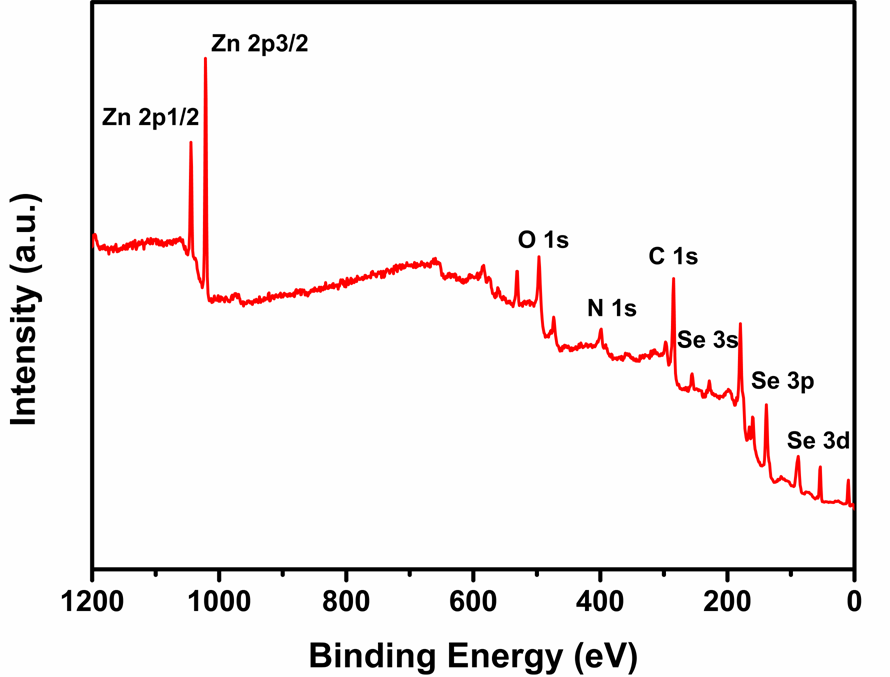


Additional file 1: Figure S3. XPS survey spectra of ZnSe/NC-300


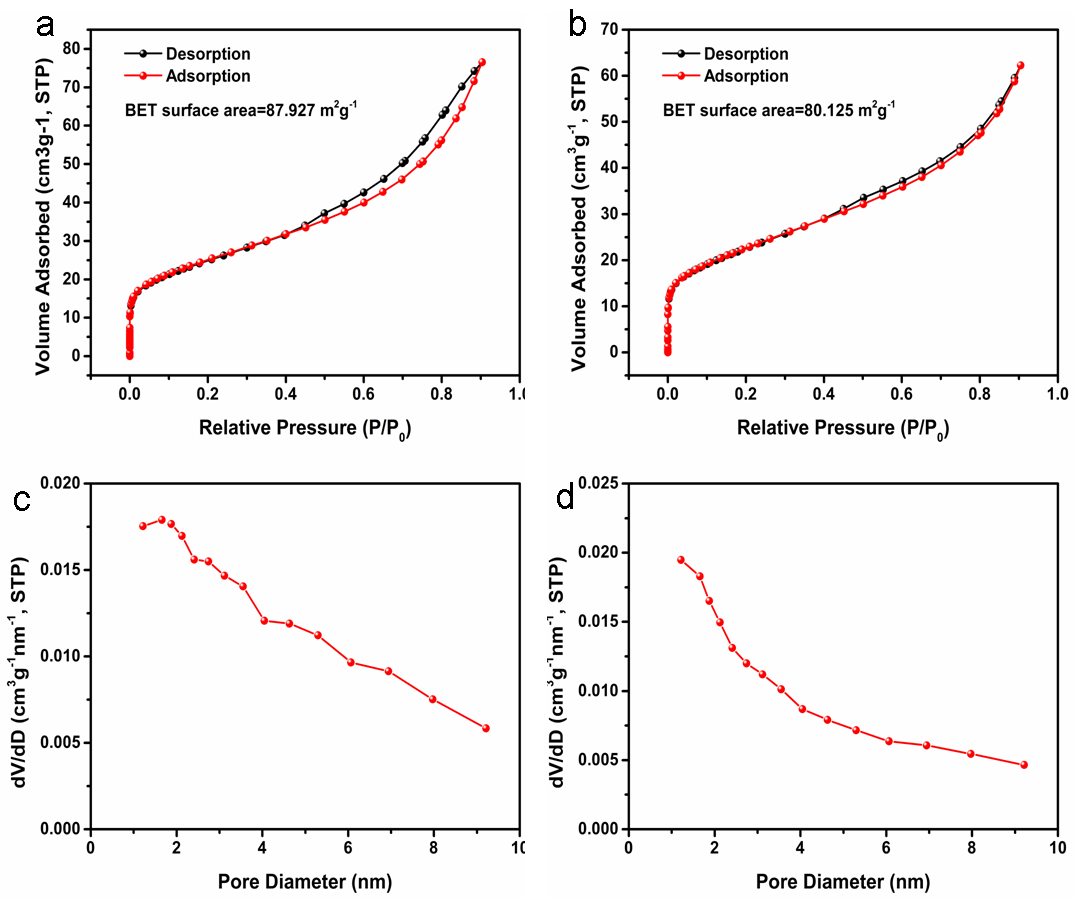


Additional file 1: Figure S4. (a,b) Nitrogen adsorption-desorption isotherms of ZnSe/NC-900 and ZnSe/NC-40, respectively, (c,d) their pore diameter distribution profiles.


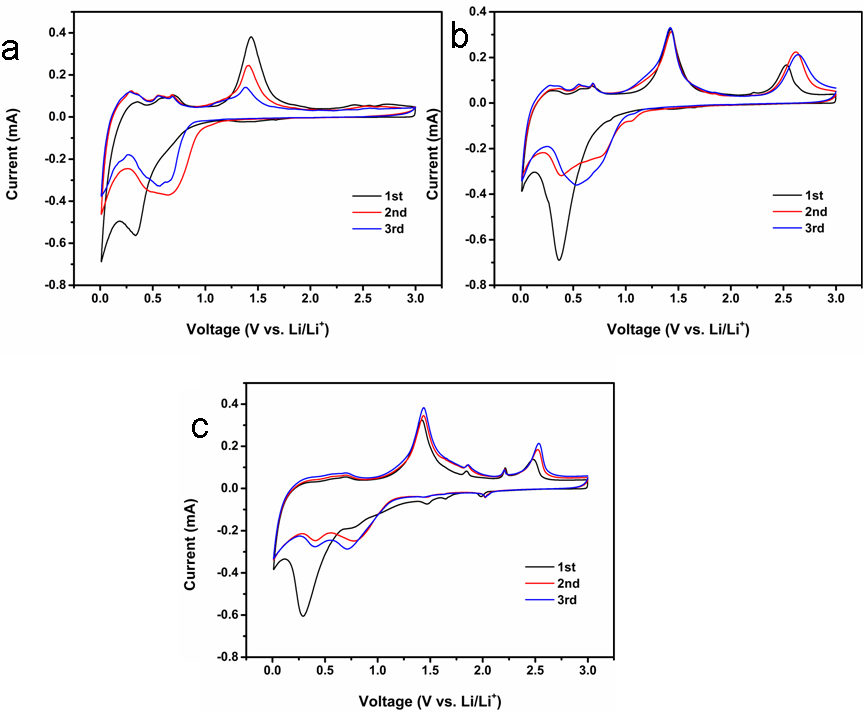


Additional file 1: Figure S5.The firs three cyclic CV cures of (a) pure ZnSe,（b）ZnSe/NC-900 and (c)ZnSe/NC-40 at a scan rate of 0.2 mV/s in the range 0f 0.01-3.0 V.


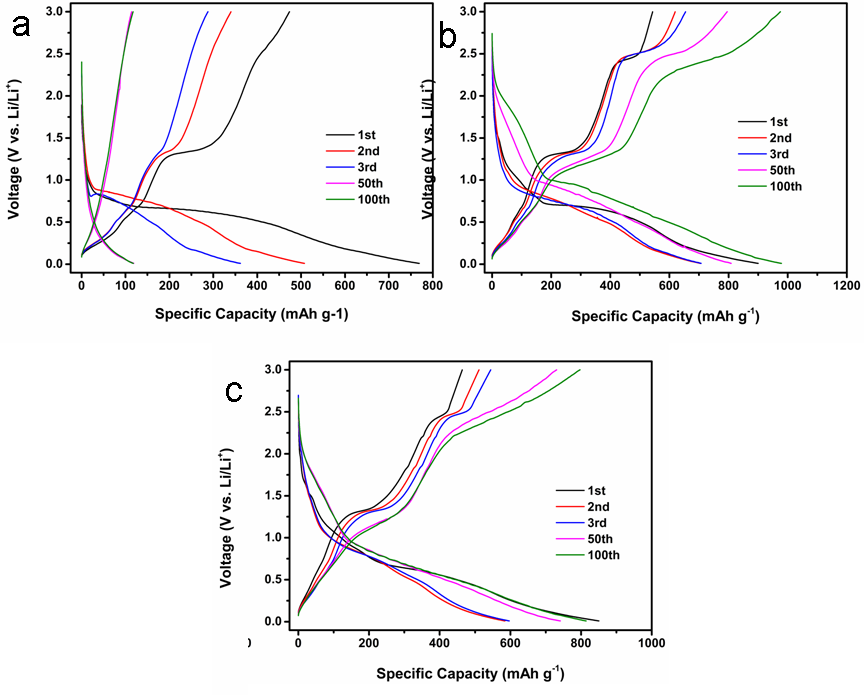


Additional file 1: Figure S6. Galvanostatic discharge/charge Voltage profiles of (a) pure ZnSe,（b）ZnSe/NC-900,（c）ZnSe/NC-40 at a current density of 100 mA g^-1^.

Additional file 1: Table S1. Comparation of ZnSe/NC composites and other metal selenides as LIB anodes

| Electrode materials | Current Density (mA/g),  Cycle Number( cycles) | Discharge capacity (mAh/g) | references |
| --- | --- | --- | --- |
| ZnSe | 100,300 | 705 |  |
| Spherical-like ZnSe | 100,50 | 433 |  |
| CoSe/C | 200,100 | 860 |  |
| SnSe-GO | 100,100 | 760 |  |
| NiSe/C | 1000,500 | 620 |  |
| ZnSe-rGO | 200,100 | 732.2 |  |
| ZnSe/NC composites | 100,100 | 947.11 | This work |
| ZnSe/NC composites | 1000,500 | 724.4 | This work |


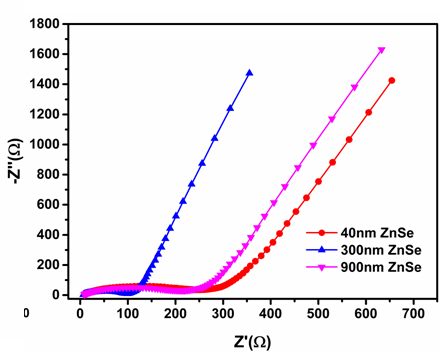


Additional file 1: Figure S7. EIS spectra of pure ZnSe, ZnSe/NC-900, ZnSe/NC-300 and ZnSe/NC-40 after 100 cycles.

**References**

[1] H.-T. Kwon, C.-M. Park (2014) Electrochemical characteristics of ZnSe and its nanostructured composite for rechargeable Li-ion batteries, Journal of Power Sources 251: 319-324.

[2] Y. Fu, Z. Zhang, R. Du, Y. Qu, Q. Li, X. Yang (2015) Spherical-like ZnSe with facile synthesis as a potential electrode material for lithium ion batteries, Materials Letters 146: 96-98.

[3] H. Hu, J. Zhang, B. Guan, X.W. Lou (2016) Unusual Formation of CoSe@carbon Nanoboxes, which have an Inhomogeneous Shell, for Efficient Lithium Storage, Angewandte Chemie-International Edition 55: 9514-9518.

[4] D. Wang, K. Zhang, Y. Zhu, Y. Lan, L. Hu, N. Lin, J. Zhou, Y. Qian (2016) A novel strategy to prepare graphene oxide-wrapped nanocrystals composite for high-performance lithium storage, Materials Letters 175: 32-35.

[5] X. Zhu, S. Li, J. Li, R.N. Ali, H. Naz, P. Liu, C. Feng, B. Xiang (2019) Free-standing WTe_2_ QD-doped NiSe/C nanowires for highly reversible lithium storage, Electrochimica Acta 295: 22-28.

[6] X. Cao, A. Li, Y. Yang, J. Chen (2018) ZnSe nanoparticles dispersed in reduced graphene oxides with enhanced electrochemical properties in lithium/sodium ion batteries, Rsc Advances 8: 25734-25744.
